# Who needs their descending thoracic aorta anyway? Extra-anatomic bypass for aorto-bronchial fistula after TEVAR

**DOI:** 10.1186/s13019-023-02326-x

**Published:** 2023-08-14

**Authors:** Joshua S. Newman, Stevan S. Pupovac, S. Jacob Scheinerman, Jui-Chuan Tseng, Jonathan M. Hemli, Derek R. Brinster

**Affiliations:** 1 Department of Cardiovascular and Thoracic Surgery, North Shore University Hospital, Northwell Health, 300 Community Drive, Manhasset, NY USA; 2 Department of Cardiovascular and Thoracic Surgery, Lenox Hill Hospital, Northwell Health, New York, NY USA

**Keywords:** Aortobronchial fistula, TEVAR, Hemoptysis, Extra-anatomic, Descending thoracic aorta

## Abstract

**Background:**

Aortobronchial fistula after TEVAR remains a vexing clinical problem associated with high mortality. Although a combination of endovascular and open surgical strategies have been reported in managing this pathology, there is as yet no definitive treatment algorithm that can be used for all patients. We discuss our approach to an aortobronchial fistula associated with an overtly infected aortic endograft.

**Case presentation:**

A 49-year-old female sustained a traumatic aortic transection 14 years prior, managed by an endovascular stent-graft. Due to persistent endoleak, she underwent open replacement of her descending thoracic aorta 4 years later. Ten years after her open aortic surgery, the patient presented with hemoptysis, and a pseudoaneurysm at her distal aortic suture line was identified on computed tomography, whereupon she underwent placement of an endograft. Eight weeks later, she presented with dyspnea, recurrent hemoptysis, malaise and fever, with clinical and radiographic evidence of an aortobronchial communication and an infected aortic stent-graft. The patient underwent management via a two-stage open surgical approach, constituting an extra-anatomic bypass from her ascending aorta to distal descending aorta and subsequent radical excision of her descending aorta with all associated infected prosthetic material and repair of the airway.

**Conclusion:**

Aortobronchial fistula after TEVAR represents a challenging complex clinical scenario. Extra-anatomic aortic bypass followed by radical debridement of all contaminated tissue may provide the best option for durable longer-term outcomes.

## Background

Aortobronchial fistula is a rare but lethal complication after thoracic endovascular aortic repair (TEVAR) [1, 2]. Although primary aortobronchial and aortopulmonary communications have been reported as a consequence of intrathoracic malignancy, penetrating aortic ulcer, or aortic trauma, the majority of cases now encountered are in the setting of a previously placed endograft [3, 4]. Given the low incidence of aortobronchial fistula, with most reports describing either small series or single cases, it is difficult to generate a generalizable algorithm for the management of this challenging condition [5]. Herein we describe our management of a patient who presented with a persistent aortobronchial fistula and evidence of superinfection, despite recent endovascular treatment.

## Case presentation

A 49-year-old female sustained a traumatic aortic transection 14 years prior, managed by a 28 mm × 10 cm Gore TAG endovascular stent-graft (Gore Medical, Flagstaff, AZ, USA). Due to persistent endoleak, she underwent open replacement of her descending thoracic aorta 4 years later with a 26 mm Terumo Vascular Graft (Terumo, Tokyo, Japan). A postoperative left diaphragmatic hernia mandated re-thoracotomy sometime thereafter.

Ten years after her open aortic surgery and two months prior to presenting to our service, the patient presented emergently to another institution with hemoptysis. A pseudoaneurysm at her distal aortic suture line was identified on computed tomography (CT), whereupon she underwent placement of a 26 mm × 26 mm × 10 cm Conformable GORE TAG Endoprosthesis (Gore Medical, Flagstaff, AZ, USA). Her hemoptysis resolved, and she was discharged on empiric broad-spectrum oral antibiotics.

**Fig. 1:**
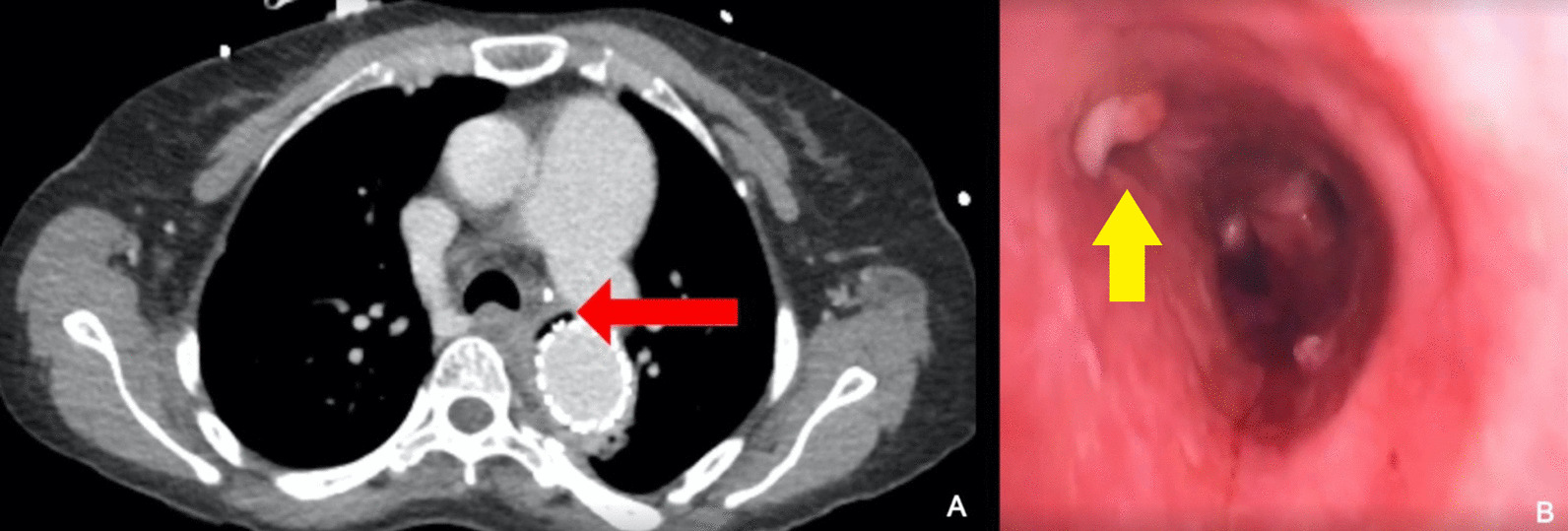
**A** CT image demonstrating air adjacent to the endograft within the descending thoracic aorta (red arrow). **B** A defect in the wall of the left main bronchus with fibrino-purulent exudate is visible at flexible bronchoscopy (yellow arrow)

Two months after her endovascular procedure, the patient presented to our institution with dyspnea, recurrent hemoptysis, malaise, and fever. CT demonstrated a pocket of gas adjacent to her thoracic endograft (Fig. 1A), findings consistent with overt graft infection. The patient subsequently had blood and sputum cultures obtained, which were found to be negative. As part of her preoperative assessment she underwent transthoracic echocardiogram (TTE), which was found to be unremarkable. Flexible bronchoscopy was performed, which identified significant fibrino-purulent exudate in the left mainstem bronchus and a fistulous connection to the mediastinum (Fig. 1B).

**Fig. 2:**
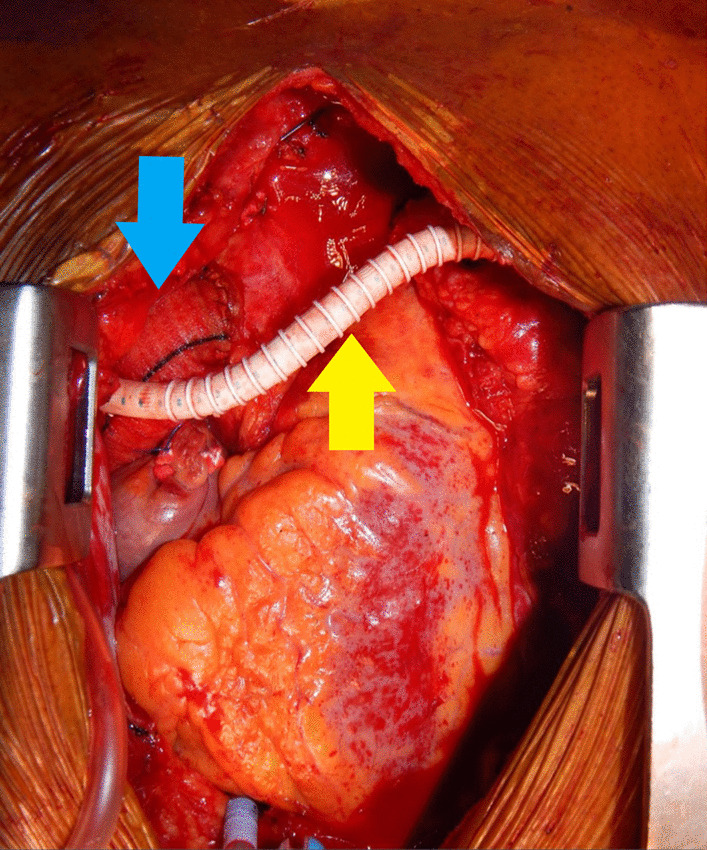
A prosthetic graft (blue arrow) extends from the ascending aorta, inferiorly around the right ventricle, to touch down at the distal descending thoracic aorta, just above the diaphragm. A separate extra-anatomic graft (yellow arrow) arises from the neo-aorta and is anastomosed to the left axillary artery

The patient was taken to the operating suite four days after presentation, where she underwent median sternotomy. Cardiopulmonary bypass was established with direct ascending aortic and dual-stage right atrial venous cannulation. Antegrade and retrograde cardioplegia cannulas were placed for the potential requirement of a cross-clamp. The heart was retracted cephalad using the suction positioning device that we routinely use in off-pump coronary artery bypass grafting (Acrobat-i Positioner, Getinge LLC, Wayne, NJ, USA). The retrocardiac pericardium was exposed and opened, facilitating access to the distal descending thoracic aorta just above the diaphragm. Intraoperative transesophageal echocardiography (TEE) estimated the descending thoracic aorta just distal to her prior repair—the site of intended anastomosis—at approximately 18 mm. A partially occluding clamp was then applied to the descending aorta, an aortotomy was made, and an 18 mm Hemashield Gold prosthetic graft was anastomosed to the aorta at this location (Getinge LLC, Wayne, NJ, USA). The prosthetic graft was looped around the inferior wall of the right ventricle, behind the supra-diaphragmatic inferior vena cava, and around the lateral wall of the right atrium to be anastomosed to the greater curvature of the ascending aorta (Fig. 2).

The aortic arch was mobilized and transected in zone 2, just distal to the left common carotid artery, using a 45 mm vascular stapler. An extra-anatomic bypass was constructed with a 6 mm Propaten graft from the ascending aorta to the left axillary artery (Fig. 2), the latter exposed via a separate left infraclavicular incision (Gore Medical, Flagstaff, AZ, USA).

**Fig. 3:**
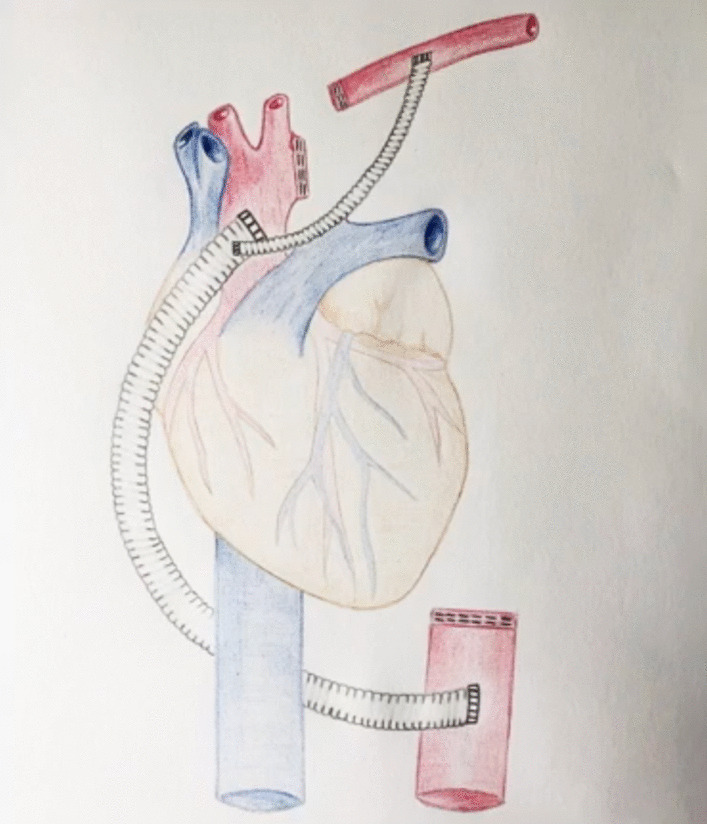
A diagrammatic representation of the patient’s new anatomy, comprising an extra-anatomic graft from the ascending aorta to the distal descending thoracic aorta, and a separate graft to the left axillary artery

**Fig. 4:**
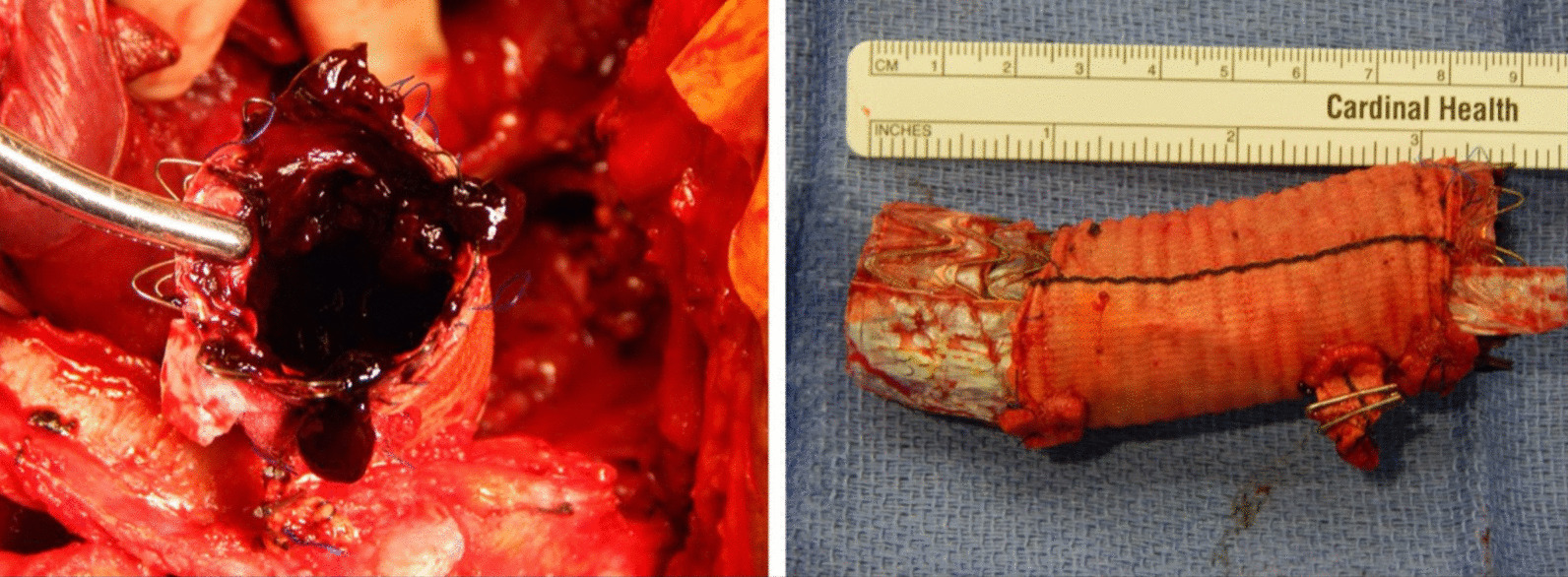
The excised descending aorta, including previous surgical graft and more recently implanted endograft within

The patient recovered well and on the fifth postoperative day returned to the operating room for the planned second stage operation. Via a reoperative left thoracotomy, almost the entirety of the patient’s descending thoracic aorta was mobilized and excised, extending from the transected arch at zone 2, to the distal aorta just above the anastomosis to the extra-anatomic prosthetic graft (Fig. 3). The excised portion of aorta included the previous surgical graft as well the endograft that had been placed more recently to control the suture line pseudoaneurysm (Fig. 4). A latissimus dorsi muscle flap was used to reinforce the posterior wall of the left main bronchus.

The patient made an uncomplicated recovery and was discharged to a rehab facility 11 days after her second operation with broad-spectrum intravenous antibiotic therapy to continue for 6 weeks thereafter. At three month follow up, she remained afebrile with no hemoptysis or shortness of breath, tolerating a diet, and recovering well.

## Discussion and conclusions

Aortobronchial fistula carries a high mortality rate, almost irrespective of the interventional strategy adopted. Temporizing the aortobronchial communication using endovascular techniques to control the hemoptysis seems to be an acceptable initial approach. An aortic endo-prosthesis is typically utilized for this purpose, as was the case in our patient, but other transcatheter occlusion devices have been employed [6]. Whether TEVAR is suitable as a long-term solution is more controversial as there are concerns related to contamination and infection of the endograft, the potential need for life-long suppressive antibiotic therapy with variable efficacy, and reported reinfection and recurrent bleeding complications [7]. In a review by Anastasiadou et al. [8] looking at management of aortobronchial fistula, they observed a 20% recurrence/infection rate, with 93% occurring in patients who were managed with an endovascular approach . Avoiding surgical intervention and pursuing medical management alone of an infected TEVAR graft has associated mortality rates exceeding 80% [9].

It is interesting that our patient presented with clinical evidence of an endograft infection so soon after her TEVAR as graft infection in the setting of aortobronchial fistula is more typically encountered as a later complication. Given the rapidity with which her septic picture evolved, we wonder whether the suture line pseudoaneurysm that she initially had was, in fact, the consequence of an existing graft infection, such that her TEVAR was implanted into a field that was already grossly contaminated.

Endovascular management of an aortobronchial fistula runs the risk of placement of a stent graft into an infected field. Therefore, we believe that definitive treatment of an aortobronchial fistula entails: radical debridement of any descending aorta that is in communication with the lung, reconstruction of the aorta, and repair of the airway. Although necessary for definitive management, this strategy has reported mortality rates in the range of 25–55% [10–13]. In-line reconstruction of the aorta may seem to be a more straightforward option than heterotopic bypass, but if this strategy is pursued one has to remain wary about potentially implanting new prosthetic material into an infected surgical field, even if antibiotic-soaked grafts, cryopreserved homograft, or self-constructed tubes of xenograft pericardium are utilized [14–16]. Our decision to utilize extra-anatomic aortic bypass afforded us the opportunity to avoid the septic area altogether, which mandated two separate operations. Although not a trivial undertaking, we deemed it the course of action most likely to ensure a durable long-term outcome. Indeed, this approach has been successfully utilized by others with varying technical modifications [17, 18].

Graft selection for an extra-anatomic aortic bypass follows similar principles to in-line replacement. In a recent review, it has been shown that the predicted size of the female descending aorta under 45 is 20 ± 3 mm [19]. Although aortic size increases with age, this is not related to increased flow demand but rather aortic remodeling with advancing age. Therefore, when sizing an extra-anatomic aortic bypass, we aim for a graft diameter of 14–18 mm, as suggested for coarctation repairs, and size match the descending aorta as appropriate. When performing extra-anatomic arterial bypasses, such as the aorto-axillary bypass in our patient, the graft is chosen based on the size of the target vessel.

It is worth mentioning that we did not employ cerebrospinal fluid drainage during the second-stage procedure whereby we removed the patient’s descending thoracic aorta. Although spinal cord ischemia is clearly a potential risk of this operation, our patient already had most of her intercostal vessels either covered or ligated because of her prior open and endovascular aortic procedures. Consequently, we did not anticipate that removing her descending aorta would entail sacrificing many new intercostal vessels that would unduly jeopardize her spinal cord. Importantly, this decision needs to be individualized for each patient.

Aortobronchial fistula after TEVAR remains a highly malignant condition that is challenging to treat. A staged approach, involving extra-anatomic aortic and great vessel bypass followed by radical debridement and removal of all infected and contaminated tissue may afford the best chance at optimal long-term outcomes.

## Data Availability

All data generated or analyzed during this study are included in this manuscript.

## Abbreviations

CT: Computed tomography
TEE: Transesophageal echocardiography
TTE: Transthoracic echocardiography
TEVAR: Thoracic endovascular aortic repair
